# Guillain-Barré Syndrome Associated with Primary Parvovirus B19 Infection in an HIV-1-Infected Patient

**DOI:** 10.1155/2012/140780

**Published:** 2012-11-06

**Authors:** Caroline Bucher Praz, Cédric Dessimoz, Frank Bally, Sitthided Reymond, Nicolas Troillet

**Affiliations:** ^1^Service of Internal Medicine, Department of Internal Medicine, Mid-Valais Hospital Center, 81, Avenue Grand-Champsec, 1950 Sion, Switzerland; ^2^Service of Infectious Diseases, Central Institute of the Valais Hospitals, 86, Avenue Grand-Champsec, 1950 Sion, Switzerland; ^3^Service of Neurology, Department of Internal Medicine, Mid-Valais Hospital Center, 81, Avenue Grand-Champsec, 1950 Sion, Switzerland

## Abstract

Parvovirus B19 (B19V) infection has rarely been reported as responsible for Guillain-Barré syndrome (GBS). We present the case of a 63-year-old man with AIDS who presented with rapidly progressing weakness of his inferior limbs and a newly appeared pancytopenia. CSF examination and electromyography were characteristic for GBS. Very high CSF and serum B19V DNA concentrations were present, in the absence of IgG or IgM against B19V. The neurologic and hematologic abnormalities improved after a 5-day course of i.v. immunoglobulins in parallel with a dramatic decrease in the B19V viral load.

## 1. Introduction

Guillain-Barré syndrome (GBS) is characterized by rapidly evolving symmetrical limb weakness, loss of tendon reflexes, absent or mild sensory signs, and variable autonomic dysfunction [1]. It occurs most commonly after a respiratory tract or a gastrointestinal infection. *Campylobacter jejuni* and cytomegalovirus (CMV) constitute the most frequent bacterial and viral triggers [1]. Epstein-Barr virus (EBV), *Mycoplasma pneumoniae*, and human immunodeficiency virus (HIV) have also been associated with GBS whereas parvovirus B19 (B19V) is not usually cited as a cause of GBS [1]. We report a case of GBS in a chronically HIV-infected patient in association with a primary B19V infection. 

## 2. Case Presentation

A 63-year-old man was admitted because of severe weakness of his inferior limbs that had worsened within a week. Ten months earlier, he had been diagnosed with advanced HIV-1 infection (relapsing bacterial pneumonia, oropharyngeal candidiasis, weight loss, CD4 lymphocytes 75 cells/mm^3^, viral load 433,413 copies/mL). Antiretroviral therapy (ART) with tenofovir, emtricitabine, and boosted darunavir had been started at this time and regularly taken since then. Three months before admission, he had suffered from vertebral fractures due to osteoporosis and had been treated with biphosphonates, calcium, and vitamin D_3_. One month before admission his CD4 count was 274 cells/mm^3^ (20% of total lymphocytes) and his HIV viral load was <20 copies/mL. At admission, he had been on dexamethasone 4 mg/day for 5 weeks, administered by his family doctor because of persisting vertebral pain. A diffuse flaccid paraparesis was present with loss of tendon reflexes, multimodal distal sensory loss, and proprioceptive ataxia. A newly appeared blood pancytopenia was present (leucocytes 1100 cells/mm^3^, hemoglobin 95 g/L, thrombocytes 25 cells/mm^3^). Creatinine and liver function tests were in the normal range. The CD4 count was 26 cells/mm^3^ (16% of total lymphocytes) and the HIV viral load 44 copies/mL. No thoracic or abdominal lesions were detected by CT scan. Electroneuromyography (ENMG) was highly suggestive of demyelinating polyradiculoneuropathy with prolonged distal motor latencies (>9 ms), decreased amplitudes of compound muscle action potential, slow nerve conduction velocities (30 m/s), and increased F-waves latencies (>63 ms), without acute denervation. No medullar lesion suggestive of any viral infection was seen by MRI of the lumbar and thoracic spine. The CSF examination showed elevated proteins (600 mg/L) without leucocytes, erythrocytes, or atypical cells. Serum IgG, without IgM, were present against CMV, varicella zoster virus (VZV), and EBV, the latter with anti-EBNA IgG. No IgG or IgM against B19V were found. Blood PCRs were positive for B19V (520 × 10^9^ copies/mL), EBV (14.4 × 10^3^ copies/mL), and CMV (4.3 × 10^3^ copies/mL). CSF PCRs were positive for B19V (290 × 10^3^ copies/mL) and EBV (1.2 × 10^3^ copies/mL). They were negative for HIV, CMV, and VZV. 

After the interruption of corticosteroids and a five-day course of intravenous immunoglobulins (IVIG) 0.4 g/kg/day, the neurological symptoms regressed and the patient could leave the hospital. Eight days after the end of the treatment, the pancytopenia had disappeared (leucocytes 7500 cells/mm^3^, hemoglobin 102 g/L, and thrombocytes 174 cells/mm^3^), serum IgG and IgM against B19V were present, and the B19V viral load was 3 log lower (537 × 10^3^/mL). It was 190 × 10^3^/mL a month later and 27 × 10^3^/mL 2 months later. Six weeks after onset of the neurologic disease, a second ENMG showed a reduction of the above-mentioned abnormalities. Three months later the patient was readmitted for fever without neurological symptoms. The B19V viremia was 3.9 × 10^3^ copies/mL and the EBV viremia 18.9 × 10^3^ copies/mL. No CMV DNA was detected by PCR. Despite extensive workout the fever remained of unknown origin until his death a month later. The autopsy revealed a peripheral T-cell lymphoma with EBV-infected lymphoblasts in the lymph nodes, liver, and bone marrow.

## 3. Discussion

According to Singer et al., HIV-associated neurological syndromes can be classified as primary HIV neurological disease, treatment-related neurological disease, and secondary or opportunistic neurological disease [2]. Because of this patient's low serum HIV viral load, his undetectable CSF HIV level, and his predominantly motor involvement, it is unlikely that he suffered from a primary HIV polyneuropathy [3]. Since his antiretroviral treatment did not include drugs that have usually been associated with neurological toxicity [3] and since his symptoms regressed despite their continuation, an ART-associated neuropathy is also improbable. 

Various etiologies are cited for secondary neurological diseases occurring in HIV-infected patients [2, 3]. They can be related to tumors or opportunistic infections. Paraneoplastic neuropathies are usually predominantly sensory and may appear with lymphomas [4, 5]. Although this patient died of a T-cell lymphoma 4 months after he presented with GBS, we do not believe that polyneuropathy and pancytopenia were caused by a still undetected lymphoma. Indeed, both neurological and hematological abnormalities developed on dexamethasone, which should have had a positive effect on a beginning lymphoma, and they regressed in parallel with the B19V viremia after the interruption of corticosteroids. CMV is the most common viral trigger of GBS but was not found by PCR in this patient's CSF. EBV, well known for causing lymphoproliferative diseases in immunodeficient hosts [6], has been reported as a potential cause of GBS, mostly in primary infection and less frequently in chronic infection [6, 7]. This patient was chronically infected by EBV with persistent viremia and presence of viral DNA in his CSF. EBV might thus have participated in his neurological disease, but, since the regression of the symptoms was linked with a decrease in B19V DNA titers whereas EBV DNA titers remained stable through the clinical history, we do not think that EBV played a major role in his polyneuropathy. 

B19V infection is common during childhood but occurs also in adults. Serum IgG against B19V are detectable in approximately 50% of 15-year-old children and 80% of elderly people [8]. B19V has a wide variety of clinical manifestations, *erythema infectiosum* (the 5th disease), arthritis, and transient aplastic crisis being the most frequent [8]. In immunodeficient hosts, persistent B19V viremia typically causes pure red cell aplasia and chronic anemia, but it has also been associated with varying degrees of neutropenia or thrombocytopenia [8]. More rarely, neurologic disease, myocarditis, kidney disease, hepatitis, and vasculitis have been attributed to B19V [8]. In 2009, Douvoyiannis et al. reviewed 81 cases of neurologic disease associated with B19V infection [9]. Most of them were children with central nervous system (CNS) manifestations such as seizures or meningitis (median age: 8 years). Peripheral nervous system (PNS) manifestations were reported in 19 patients with a median age of 29.5 years (range 16 months–49 years). These PNS manifestations included brachial plexus neuropathy (8 cases), carpal tunnel syndrome (6 cases), cranial and peripheral neuropathies (4 cases), and a single case of GBS in a 4-year-old boy [9, 10]. To our knowledge, 3 other cases of GBS or GBS variant have been reported in association with B19V infection, in 2 adults [11, 12], and 1 child [13]. None of them had altered immunity. 

Our patient presented with an acute demyelinating polyneuropathy. Major biological features were pancytopenia, albuminocytologic dissociation, high levels of B19V DNA in serum and CSF during the acute phase in the absence of B19V IgG and IgM, and decrease of the viral load after a 5-day course of IVIG concomitant with clinical improvement. As most of the immunodeficient patients in the Douvoyiannis' review [9], our patient's neurological disease was not associated with other symptoms of B19V infection such as flu-like syndrome, rash, or arthritis. This could have been due to HIV infection and corticosteroids, both impairing his inflammatory response. 

In conclusion, this case illustrates the multiple factors that can contribute to peripheral nervous system manifestations in immunodeficient patients. It emphasizes the importance of looking for infection with neurotropic viruses by PCR in such situations and adds to the current evidence that parvovirus B19 infection may cause acute demyelinating polyneuropathy that can be improved by IVIG.
